# Aspirin Preventing Occlusion after Coil Migration into the Distal Anterior Cerebral Artery

**DOI:** 10.7759/cureus.7797

**Published:** 2020-04-23

**Authors:** Claudia L Craven, Sophie Mullins, Adam Rennie, Ahmed K Toma

**Affiliations:** 1 Neurosurgery, National Hospital for Neurology and Neurosurgery, London, GBR; 2 Neuroradiology, National Hospital for Neurology and Neurosurgery, London, GBR

**Keywords:** aneurysm, endovascular coiling, coils, intraluminal migration, asymptomatic, complication, antiplatelet

## Abstract

Endovascular coil embolization of cerebral aneurysm is a common procedure for managing cerebral aneurysms. We present a rare case of immediate silent coil migration into the pericallosal artery, without distal occlusion, following successful embolization. Despite the entire coil sitting within the lumen of the artery, good distal flow was observed. The patient remained asymptomatic throughout and had a good long-term (three years to date) outcome (modified Rankin Scale score of 1). We believe that the novel protective factor was the routine use of a single anti-platelet post-procedure.

## Introduction

Endovascular coil embolization of cerebral aneurysm is a common procedure for managing cerebral aneurysms. Endovascular coil migration is a potentially devastating event after endovascular embolization of cerebral aneurysms, often resulting in infarction and neurological deficit [1]. Migration can either occur intra-procedurally or be delayed after closure [2]. Coil migration has been reported in 0.3-6% of cases [2]. However, there are only nine case reports on silent coil migration (ranging from three days to six months) [2-4]. We present a rare case of immediate silent coil migration into the pericallosal artery, without distal occlusion following successful embolization.

## Case presentation

A 62-year-old female with a history of type 2 diabetes, hypertension, and high cholesterol presented with sudden onset headache. She had no family history of intracranial pathology or connective tissue disease and was a non-smoker.

**Figure 1:**
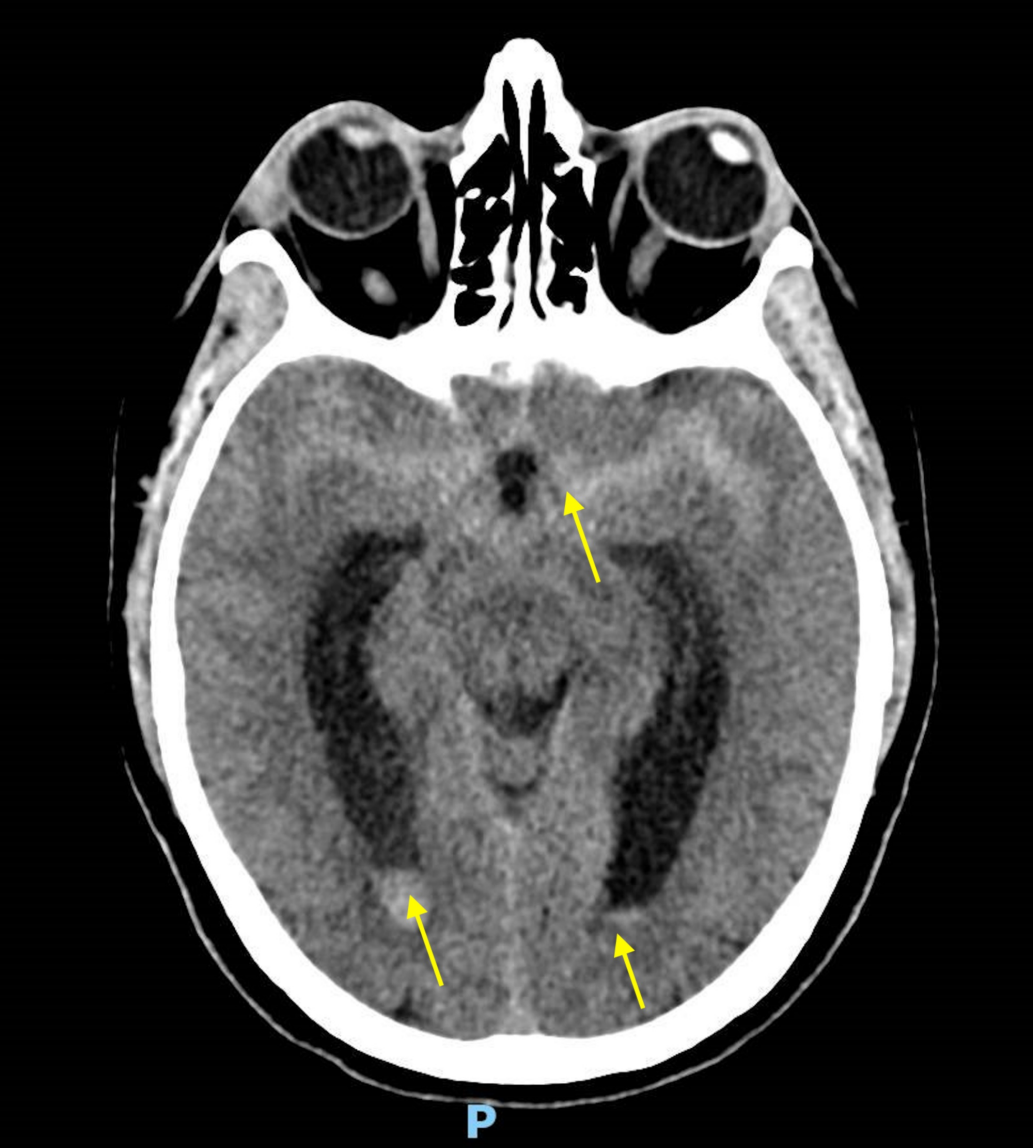
CT imaging demonstrated a subarachnoid hemorrhage of Fisher grade 4 (yellow arrows demonstrate hypodense regions of subarachnoid and bilateral intra-ventricular blood).

CT imaging demonstrated a subarachnoid hemorrhage (SAH) of Fisher grade 4 (Figure 1).

**Figure 2:**
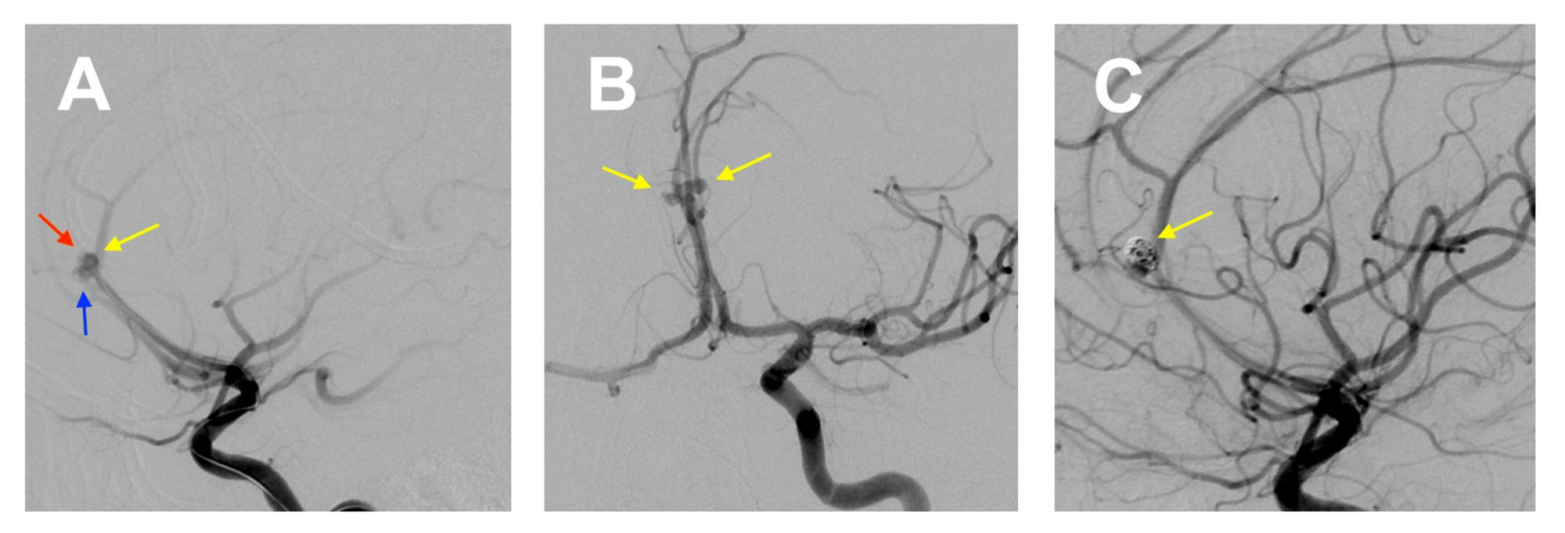
(A) DSA showing a large right saccular pericallosal aneurysm (yellow arrow), a smaller right pericallosal aneurysm (blue arrow), and a left pericallosal aneurysm (red arrow). (B) DSA demonstrating pericallosal mirror aneurysms (yellow arrows). (C) DSA showing coil embolization of the large ruptured right aneurysm (yellow arrow) DSA, digital subtraction angiography

Digital subtraction angiography showed a large right saccular pericallosal aneurysm (fundus-to-neck ratio of 5:3), a smaller right pericallosal aneurysm, and a mirror left pericallosal aneurysm (Figures 2A, 2B). Due to the presence of multiple aneurysms (cloverleaf arrangement), the procedure was performed in two stages. The two right-sided aneurysms (including the ruptured aneurysm) were treated first, and the remaining (left-sided) mirror aneurysm was treated as a second-stage procedure. Coil embolization of the ruptured aneurysm took place under general anesthesia, with two 1.5 x 2 mm three dimensional (3D) Axium™ coils (Medtronic, Minneapolis, MN, USA) being placed into the large right pericallosal aneurysm and one 1.5 x 2 mm 3D Axium™ coils being placed in the smaller right pericallosal aneurysm. The final angiogram showed good placement of the coils (Figure 2C).

During the procedure, 5,000 units of heparin and 300 mg of aspirin were given. Post-procedure, the patient remained on 75 mg of aspirin daily.

**Figure 3:**
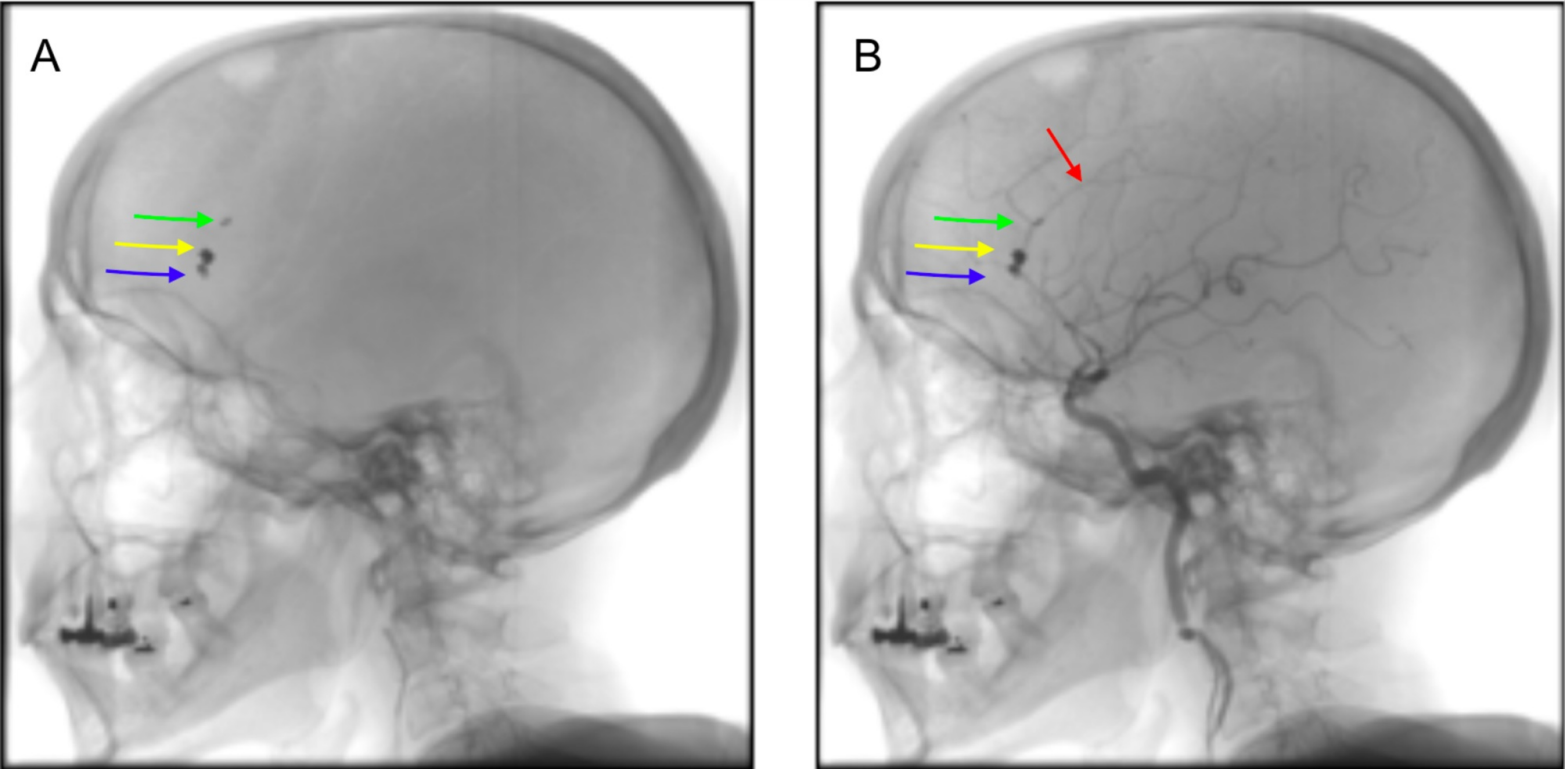
(A) Angiographic evidence of coil migration into the pericallosal segment (green arrow). Coils remain in the large right aneurysm (yellow arrow) and in the smaller right aneurysm (blue arrow). (B) DSA showing good distal flow maintained (red arrow). DSA, digital subtraction angiography

The second coiling for the left (unruptured) pericallosal aneurysm was performed six days later. During the second angiogram, it was noted that one of the coils (from the larger aneurysm coiling procedure performed six days prior) had migrated into the pericallosal artery (Figure 3A). The single coil placed into the smaller right adjacent aneurysm had not migrated (Figure 3A). Despite the entire migrated coil sitting within the lumen of the artery, good distal flow was observed (Figure 3B). The patient remained on 75 mg of aspirin daily for three months (as was routine practice at this single center at the time of intervention).

**Figure 4:**
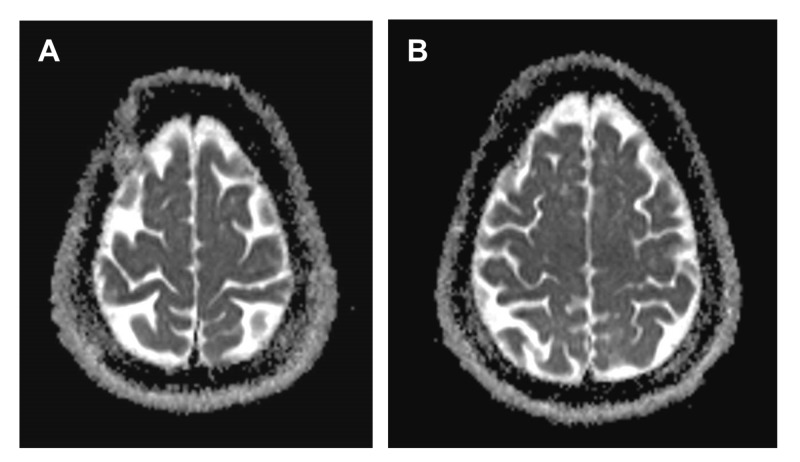
(A and B) MRI showing normal ADC in the cortical regions supplied by anterior circulation. MRI, magnetic resonance imaging; ADC, apparent diffusion coefficient

The previously ruptured aneurysm recurred and has since been secured with surgical clipping (two years post-procedure). The patient had a good long-term outcome (modified Rankin Scale score of 1 at three years after follow-up), with no motor, speech, or cognitive deficits. Magnetic resonance imaging (MRI) confirmed no evidence of infarction (Figures 4A, 4B). This patient was known to have a normal clotting profile prior to the procedure and had no known intrinsic protective factors. The patient remains asymptomatic.

## Discussion

Cases of coil migration are difficult to manage and may involve complex coil retrieval techniques. We present a case whereby the coil migration was silent, potentially owing to the standard use of post-procedure prophylactic aspirin.

Factors thought to increase the risk of coil migration include (a) coil related factors such as instability of the coil within the aneurysm, over- or under-packing of the coil, and type of coil, and (b) aneurysmal factors such as size, shape, and neck width relative to the fundus, the wall shear stress, and vortical flow within the aneurysm [3,5]. Dehydration, vasospasm, and the pro-thrombotic nature of SAH all increase the risk of distal occlusion after coil migration [3].

Concomitant stent placement has been advocated previously in cases with higher risks of coil migration [1,3]. However, in this case report, there were no features suggesting a high risk of coil migration (with the aneurysm being saccular and a not too wide neck) and no clear indication for the placement of a stent.

A similar case report of coil migration into the pericallosal segment attributed the preservation of distal flow to spiral shape of a two-dimensional ultrasoft helical coil [6]. However, here we report the maintenance of distal flow, even when using 3D coils.

Multiple different approaches to manage migrated coils - from medical to surgical - have been reported [2]. Kamide et al. reported that incidental use of the factor X inhibitor, rivaroxaban (for atrial fibrillation), may have been an important protective factor in preventing distal occlusion in their case report of delayed coil migration (identified at six months post-procedure) [3]. This case of immediate coil migration is novel because it appears to be silent, with good distal flow, due to the continuation of prophylactic aspirin post-procedure.

Studies have shown no overall benefit or increased risk with the routine use of anti-platelet therapy [7]. Many patients who undergo endovascular coiling of a cerebral aneurysm do not receive post-intervention anti-platelet therapy routinely.

However, there are reports suggesting that aspirin may be associated with reduced rates of thromboembolic events post-coiling [8,9]. The proposed mechanism of activity is both through the direct antiplatelet effect of aspirin and through reduction of the inflammatory response associated with perivascular blood [8]. Peri-procedural anti-platelet therapy is therefore standard in our center following coiling.

In our reported case of immediate coil migration, the individual was on a once-daily dose of single anti-platelet, (aspirin 75 mg). Given the lack of other protective factors (use of 3D coils and the lack of other post-procedure medications such as factor X inhibitor and normal clotting profile), aspirin appears to have been the main protective mechanism preventing occlusion. Despite having a coil sitting within the entire lumen of a small artery, the patient remarkably did not develop any occlusion.

## Conclusions

We conclude that maintaining patients on regular aspirin post-procedure could protect against the significant ischemic consequences following coil migration. Furthermore, in certain cases, medical management with regular aspirin may have a lower risk profile than alternative salvage methods such as pursuing the coil using retrieval techniques.
